# Risk Factors for Developing Rheumatoid Arthritis in Patients With Undifferentiated Arthritis and Inflammatory Arthralgia

**DOI:** 10.3389/fmed.2021.668898

**Published:** 2021-06-15

**Authors:** Marta Novella-Navarro, Chamaida Plasencia-Rodríguez, Laura Nuño, Alejandro Balsa

**Affiliations:** Rheumatology Department Hospital Universitario La Paz, Madrid, Spain

**Keywords:** rheumatoid arthritis, undifferentiated arthritis, clinical suspected arthralgia, pre-clinical phase in rheumatoid arthritis, modifiable risk factors

## Abstract

Currently, there is an increasing interest in treating patients at risk of rheumatoid arthritis (RA) to prevent the development of this chronic disease. In this sense, research has focused attention on the early identification of predictive factors of this disease. Autoantibodies and markers of systemic inflammation can be present before clinical arthritis and RA development. So, the phase of inflammatory arthralgia preceding clinical arthritis is an important part of the window of opportunity and, starting treatment might prevent progression to chronic arthritis. Additionally, the early diagnosis and treatment initiation, in patients with inflammatory arthritis at risk of persistence and/or erosive progression, are fundamental because may allow optimal clinical responses, better chances of achieving sustained remission, preventing irreversible organ damage and optimizing long-term outcomes. This review aims to give an overview of clinical risk factors for developing RA, both in suspected arthralgia and in undifferentiated arthritis. Besides taking into consideration the role of serological markers (immunological and acute phase reactants) and clinical features assessed at consultation such as: articular affection and patient's clinical perception. Other features as sociodemographic and environmental factors (lifestyle habits, microbiota, periodontal disease among others), have been included in this revision to give an insight on strategies to prevent development of RA and/or to treat it in early stages.

## Introduction

Rheumatoid arthritis (RA) is a chronic inflammatory joint disease characterized by chronic synovitis, joint destruction and disability (1). As knowledge about the physiopathology underlying RA has grown, so has the understanding of its development, even from its earliest stages, the so-called “pre-clinical” stages. It is known that the pre-clinical phase of RA development can last for years, which has led in recent years to a renewed determination to identify those patients at high risk of developing persistent joint disease, which can lead to irreversible joint damage (2). While signs or symptoms can initially be absent, autoimmunity can still exist (3). In addition, during this early phase, arthralgia without clinical synovitis may be present, or patients may develop inflammatory arthritis without fulfilling any of the classifiable forms of arthritis (2).

For these reasons, Early Arthritis Clinics (EACs) have been progressively implemented in rheumatology departments in order to treat patients with early inflammatory joint disease as well as those with clinically suspect arthralgia (CSA) (4). Stratifying and identifying these patients early on is of great importance, particularly since the concept of a “window of opportunity” (5) has now been firmly established, one in which attitudes toward therapy at the onset of disease can lead to better treatment outcomes.

In these early stages, patients can present clinically suspected arthralgia (CSA) or undifferentiated arthritis (UA). The European League Against Rheumatism (EULAR) (6) established a set of clinical characteristics for clinical suspected arthralgia in an attempt to identify the risk of progression to RA. UA is a form of arthritis that does not fulfill the classification criteria for RA or other inflammatory arthritis (7).

The available data indicate that patients with CSA constitute a small percentage of those with arthralgia attended in rheumatology departments, approximately 7% (8). However, about 20% of patients initially presenting CSA will progress to RA (9). Regarding UA, the progression rate to RA occurs in approximately one third of patients (7, 10). Since these data show that a considerable percentage of patients can progress to RA, it becomes even more important to identify them early on. Furthermore, early identification affords not only the possibility of prevention, but also testing the efficacy of pharmacological interventions in those patients at risk of developing RA. Several trials are ongoing or have recently been completed (11–17). Nonetheless, a common issue hampering such trials is the great difficulty of patient recruitment. Once again, to improve inclusion in trials, strategies should be implemented, to include optimizing education about RA, personal risks, trial aims and medications, and explicitly addressing any patient concerns; see the recently published results by van Boheemen et al. (18).

For this narrative review, we conducted an exhaustive search of PubMed, limiting the search interval to the most relevant and recent articles. Review articles, recommendations, longitudinal studies and clinical trials were retrieved in order to summarize and synthesize the information obtained. We focused on risk factors for the onset of RA in susceptible patients (CSA or UA) and evaluated modifiable factors that can affect disease development.

## Clinical Factors

Clinical features that may help predict the progression to RA, especially in those patients presenting arthralgia, represent a challenge for rheumatologists. Musculoskeletal symptoms are very prevalent in the general population and arthralgia constitutes a non-specific symptom as the nature of the pain can be diverse (19). Most arthralgia patients referred to rheumatologists have been diagnosed with something other than RA. Nonetheless, it is important to evaluate such patients and try to classify them as being at low or high risk of RA progression. The EULAR task force established a set of seven clinical characteristics for patients at risk for RA: joint symptoms of recent onset (duration < 1 year); symptoms centered on metacarpophalangeal (MCP) joints; stiffness duration ≥ 60 min; most severe symptoms are present in the early morning hours; presence of a first-degree relative with RA; difficulty making a fist and successfully completing a positive squeeze test. The presence of ≥3 parameters yields great sensitivity and ≥4 parameters represents great specificity (6).

Other studies have proposed slightly different risk factors, such as the presence of metatarsophalangeal or metacarpophalangeal involvement, or morning stiffness >30 min (20). On the other hand, data from patients with seropositive arthralgia revealed that intermittent symptoms, a visual analog scale (VAS) ≥ 50 and the location of symptoms in both the upper and lower extremities were all predictors of RA development (21). Regarding morning stiffness, van Nies et al. showed in a multicenter cohort study that almost all arthralgia patients without arthritis did not experience morning stiffness for ≥90 min. Thus, this parameter seems to have a very good negative predictive value (22).

Data suggest that serological and/or imaging features are needed to correctly classify these patients (23). In addition, more studies should be carried out to corroborate the above-mentioned findings in other cohorts.

With regard to UA, the identification of symptoms as possible predictors of progression to RA seems a priori easier than in the case of CSA. For this reason, numerous studies have proposed clinical factors predicting the transition from early UA to RA (10, 24–27).

Regarding joint involvement, several studies agree that polyarticular and symmetric involvement are related to RA development (10, 24–27). Moreover, the number of joints affected and the location of the initial joint symptoms have been described as risk factors. Synovitis in the upper extremities, the involvement of small joints as well as both small and large joints, imply a greater risk of RA than when limited to only the lower extremities or large joints (10, 25–27). However, Brinkmann et al. found that swollen shoulder joints are an independent risk factor (24).

The duration of symptoms until referral to a rheumatologist is also important. Although in most EACs symptom durations <1 year are an inclusion criterion, patients with gradual symptom onset had a 1.5 higher odds ratio (OR) of developing RA than those with acute or subacute symptoms (10, 25–27). In studies performed with patients diagnosed with very early arthritis (i.e., disease durations < 16 weeks), symptom duration did not show any relation to progression to RA (24, 28).

Morning stiffness has been evaluated both for its duration and intensity and studies have shown that morning stiffness durations ≥60 min, as well as the severity of morning stiffness as measured by VAS, both correlate with, and constitute a predictive factor for, developing RA (17, 18).

## Epidemiological Factors

Epidemiological factors include age, sex, family history of RA and race/ethnicity (29). However, variability in the study designs, outcome definitions, serological subsets and different follow-up times can hamper the ability to obtain robust conclusions (18, 30, 31).

### Sex

Approximately two-thirds of individuals who develop RA are women (29). The cumulative risk of developing RA in adult population has been estimated at 3.6% for women and 1.7% for men (32). The role of hormones in the development of RA remains controversial, but this higher frequency of RA in women may be attributed to the stimulatory effects of estrogens on the immune system (33). Factors that have been associated with an increased risk for on the onset of RA include early menopause (34), the presence of polycystic ovary syndrome (35), pre-eclampsia (36), and post-partum periods (37, 38).

Factors that can protect against the onset of RA include breast-feeding (34, 39), the use of hormone replacement therapy and oral contraception (40). Oral contraceptive use has been associated with decreased rates of Rheumatoid Factor (RF) positivity in first-degree relatives (FDRs) of patients with RA, suggesting there may be an early “pre-clinical” effect of hormones on the pathogenesis of RA (41). The mechanisms involved in the relationship between hormone use and decreased risk for RA remain still unknown, although exogenous hormones may lead to decreased endogenous hormone production, which implies a risk reduction (42). The data on other factors, such as parity, are more conflicting (43, 44). One study showed no increased risk for seropositive RA on the basis of one or more pregnancies (44) while others, including a meta-analysis, demonstrated that pregnancy confers a protective effect (43, 44).

### Family History of RA

There is an increased prevalence of RA in those families with an ~40–50% risk for seropositive RA, being strongest in first-degree relatives (FDRs) (45). Several studies have evaluated the risk of RA development in FDRs (46–48). For example, Barra et al. found that the rate of circulating anticyclic citrullinated peptide antibody (ACPA) positivity in the unaffected FDRs of RA patients with a high prevalence of the shared epitope (SE) and smoking was 48%, whereas ACPA was rare among healthy controls. One study conducted in 1,780 FDRs of RA patients reported that screening for auto-antibodies can identify RA-related positive individuals; i.e., those who are associated with a higher risk for future onset of RA (46). In this line, another study observed a greater association of obesity, ACPA positive status and incidence of periodontitis in FDRs than in healthy controls, suggesting the relevant role of these conditions as a risk of developing RA in FDRs (49).

## Environmental Factors

The existence of a genetic component in the onset of RA has long been considered because of the increased risk in first-degree relatives of patients with RA (50, 51). However, the overall risk associated with established genetic markers remains limited, and the relatively low penetrance of the disease hints that environmental factors play an important role in the etiology of RA (52). There is evidence supporting the possibility that autoimmunity in RA is of extra-articular origin, given the fact that ACPA and/or RF can be found up to a decade prior to disease onset. Moreover, antibody titers, epitope specificity and circulating proinflammatory cytokines increase during the pre-clinical phase (53–57). Recently, attention has focused on the interactions of environmental and genetic factors in the pathogenesis of RA (58). However, some of the results are controversial because they may be related to heterogeneity in exposure definitions, study designs, and populations studied.

### Gut Microbiota

The gut microbiota co-develop with the host from birth, containing a vast array of bacteria that participate in digestion, nutrition, vitamin synthesis and metabolic functions. The composition and functions of the gut microbiota play a key role in regulating the balance of Th17/Treg cells in the lamina propria, influencing in host immune responses, tolerance, and susceptibility to such autoimmune diseases as RA (59, 60).

Recent evidence supports the hypothesis that RA autoimmunity may originate in the mucosal immune system through local autoimmune processes (61–63). Gut microbiota could induce Th17 cell differentiation via multiple pathways, inducing the secretion of various pro-inflammatory cytokines, which have been implicated in the pathogenesis of RA. The production of short-chain fatty acids resulting from the microbial fermentation of dietary fibers, such as butyrate, acetate and propionate, is one of the proposed pathways by which intestinal microbiota affect Treg cell differentiation and systemic inflammation (64). Another mechanism that may explain the influence of gut microbiota on the appearance and perpetuation of inflammatory diseases is the modulation of intestinal permeability (65). Intestinal barrier strength and function can be affected by some factors, including diet, smoking habit, gut microbiota composition and mucosal immune system integrity. When the mucosal barrier is disrupted, the exogenous luminal constituents invade intestinal cells and can trigger an abnormal immune response. This can result in local and systemic inflammation which increase the risk of developing both gut-associated and extra-intestinal diseases (66).

The presence of bacteria in mucosal surfaces is sufficient to alter local and systemic host immune responses and to elicit joint inflammation in animal models (67). Host genetics may constitute a predetermined factor for harboring a unique microbiome profile, especially *Prevotella* spp. (68, 69). Cases of early RA onset have been shown to have a higher diversity of fecal lactobacillus communities (70). Intestinal dysbiosis associated with RA is not limited to a disbalance of Lactobacillus. In particular, Haemophilus species become depleted and *Prevotella copri* are more abundant in fecal samples from new-onset RA patients than in healthy controls (71). Notably, the prevalence of Haemophilus species has a negative correlation with autoantibody levels, raising the hypothesis that the bacteria may play a protective immune-modulating role (72).

The regulation of gut microbiota through nutritional factors is increasingly recognized as a potential interventional strategy for the prevention and management of inflammatory diseases such as RA. In humans, regular adherence to a Mediterranean diet modulates gut microbiota and increases short-chain fatty acid production, while subjects with lower adherence to such a diet tend to have increased intestinal permeability (73, 74). Although a number of studies have suggested associations between dietary habits, mainly regarding salt, fruit, vegetable and/or meat intake, and RA development, the results remain inconclusive (75, 76).

In summary, dysbiosis could predispose some individuals to RA due to both the excessive growth of pro-inflammatory species and the depletion of protective species with immunomodulatory functions. It is now recognized that both genetic factors and acquired conditions (dietary and health habits, social status, exposure to pathogens, and drugs) can dynamically contribute to the composition of the microbiota.

### Dietary Factors

Diet may act either as a disease trigger or a “moderator” through foods that either exacerbate or reduce inflammation (77). Although several foods and diet types have been evaluated in terms of their possible links to the risk of RA, the evidence remains controversial due to the difficulty of determining their individual impact.

A diet rich in dietary sodium (salt) has been associated with an increased risk of RA (75). High salt intake could potentiate the effect of other environmental factors, such as smoking, thereby increasing the risk of ACPA positivity and the differentiation of Th17 (78, 79).

An association between sweetened drinks and RA development has also been described. England's National Health Service (NHS) found that regular consumption of sugar-sweetened soda, but not diet soda, was associated with an increased risk of seropositive RA (80). In addition, high-fructose soft drinks have been linked to arthritis in young US adults (81).

Excessive consumption of red meat has been correlated to an increased risk of inflammatory polyarthritis (82). An intake of >88 g red meat/day, compared to <49 g/day, doubled the risk of developing inflammatory polyarthritis (OR: 2.3, 95% CI: 1.1–4.9; *p* = 0.03) (82). However, a multicenter Chinese case-control study that monitored the dietary patterns of 968 RA patients and 1,037 healthy controls for 5 years prior to clinical onset of the disease found no differences statistically significant in red meat consumption between the two groups (83).

Coffee consumption has traditionally been accepted as a risk factor of RA. A recent meta-analysis of coffee suggests that consumption ≥4 cups coffee/day increases risk of seropositive, but not seronegative, RA (84). These results should be interpreted with caution since other potential confounders—e.g., smoking, stress and lower dietary quality—can influence the amount of coffee consumed. This same meta-analysis found that consumers of ≥3 cups of tea per day presented a reduced risk of RA (84).

Different studies analyzed Omega-3 fatty acids as possible protective factors for RA. A Danish prospective cohort study observed a decreased risk in patients with a high dietary intake (>8 g fat/100 g fish), while a medium consumption of fatty fish (3–7 g fat/100 g fish) was associated with a significantly higher risk of RA (85). In women, a dietary intake of long-chain n-3 polyunsaturated fatty acids >0.21 g/day was associated with a 35% decreased risk of developing RA compared with a lower intake (86). Furthermore, in FDRs of patients with RA, it was found that increased intake of fatty acids were associated with a lower risk for RF and ACPA positivity (87, 88). Notably, there appeared to be a greater protective effect in those individuals positive for at least one SE containing allele, suggesting that there may be an interaction between these factors in mediating RA risk (87). However, the EIRA study, a population-based case-control study evaluating more than 4,000 participants, demonstrated only a mild decrease in RA risk with regular consumption of fatty fish (89).

The role of fruit and vegetables on RA risk has also been studied. The EPIC-Norfolk study found a higher intake of fruits and vitamin C in healthy controls compared to patients with inflammatory arthritis, and a decreased risk of RA development in olive oil consumers (90). Possible mechanisms that might explain such findings include the antioxidant properties of fruit and vegetables as well as the natural tocopherols in olive oil that act as radical scavengers (91). On the other hand, the previously described Danish prospective study did not find any associations between RA and intakes of vegetables, citrus, r, beta carotene, retinol, zinc, iron, selenium vitamins A, E, C, D, and/or meat (85). The reasons for these null findings are not clear, but may relate to the overall dietary patterns (types and amounts of fruit/vegetables consumed).

The data concerning alcohol intake and its impact on the risk of RA remains contentious. In terms of alcohol, consumption of 3–5 standard drinks/week has been associated with 22–31% reduced risk of developing RA compared to complete abstention (92). In their multivariate analysis a Dutch longitudinal study involving 374 individuals who initially presented arthralgias, ACPA and/or RF positivity, though without inflammatory arthritis at baseline, found that alcohol intake resulted a protective factor in the development of RA (21). In contrast, in a British study of 100 ACPA-positive subjects, alcohol consumption was not significantly protective (93). Moreover, additional studies will need to be developed in order to elucidate the role of alcohol in the risk and prevention of RA.

Currently, the data on milk and dairy products are controversial, with no discernable association or protective effect, which is probably attributable to its vitamin D content (94).

While vitamin D deficiency has a great prevalence in patients with autoimmune diseases, including RA, how and whether low serum 25(OH) D contributes to the risk of RA is less understood. Indeed, studies have reported conflicting results (95–98). The Women's Health Initiative randomized clinical trial demonstrated an increased risk of RA as a result of higher levels of vitamin D intake (97). One retrospective study involving patients with RA who had donated blood up to 5 years before disease onset found no differences in vitamin D levels between patients and healthy controls (95). In addition, no association was found between the vitamin D deficiency and the development of RA (95). However, a meta-analysis conducted on more than 200,000 subjects reported an association between RA risk and low vitamin D intake (98). These varying results reflect the limitations associated with vitamin D studies.

In general, healthy lifestyles, including the healthier diets (diets high in fruits, whole grains, and vegetables and low in sugar and animal fats) have been associated with a decreased risk for RA (83, 99, 100). In addition, included in the healthy lifestyle habits we cannot forget the influence of body mass index (BMI), which deserves to be discussed in a the following section.

### Obesity

In recent years, knowledge of the inflammatory mechanisms at work in obesity has steadily increased. White adipose tissue (WAT) is now postulated to act as an endocrine organ capable of releasing cytokines (such as C-reactive protein, leptin, tumor necrosis factor, and interleukin 6), which may predispose individuals to autoimmune inflammatory diseases by exacerbating inflammation (30). Thus, many studies have focused on assessing the association between obesity and the risk of developing inflammatory diseases such as RA.

Although data on the relationship between obesity and the risk of is conflicting, obesity is an important and increasingly common comorbidity, even on first presentation of RA (101). Increasing evidence that the period of pre-clinical inflammation and autoimmunity can be prolonged suggests that it is of pressing interest to more fully take into account obesity and other environmental risk factors that are potentially modifiable and which may therefore help lower the risk of developing RA in susceptible patients.

The results of several meta-analyses, mostly consisting of retrospective studies, support the contention that BMI is directly related to the risk of developing not only RA, especially in female populations, but also of seronegative RA in particular (102–104). However, by focusing on studies conducted when patients are in a pre-clinical period or on those with UA, the influence of obesity in the onset of RA remains controversial and only partially understood.

Rakihe et al. in a prospective study of 121 patients (100 presenting unspecific musculoskeletal symptoms and 21 with inflammatory arthritis) found no association between body mass index (BMI), ACPA positivity and the development of RA (93). However, in another case-control study, that also evaluated the influence of obesity in patients with pre-arthritis vs. controls, Tedeschi et al., correlated BMI and ACPA positivity with the development of RA, especially in women. These results suggest that ACPA and obesity can act synergistically, constituting a risk factor for the development of RA, as well as Chaparro-Sanabria et al. (105). These results also suggest that women with both risk factors are likely to progress to clinical development of RA most rapidly of any population (106).

In a prospective study involving RA patients without lacking RA-specific antibodies, De Hair et al. found that being overweight was associated with the onset of arthritis (107).

As mentioned above, elevated BMI and ACPA are both associated with heightened RA risk, particularly in women. Nevertheless, in these studies, a cross-sectional relationship between BMI and the presence of ACPA was not found. Thus, excess adipose tissue does not seem to promote the development of ACPA, although being overweight and obesity can foster systemic inflammation, which hastens the pathogenesis of RA. Building on these findings, other studies aimed to assess the role played by BMI in the development of ACPA-positive RA. Thus, Moreno-Fresneda et al. and Lu et al. found that being overweight or obese leads to ACPA-negative RA more frequently than in patients of normal weight. In conclusion, obesity is a potentially modifiable risk factor that may contribute to the onset of clinical arthritis due to exacerbation of the pro-inflammatory status in these patients (108, 109).

### Lung Mucosa

The lung parenchyma has been described as a possible site of onset of autoimmunity in RA, although the role of the pulmonary microbiome as a mediator of inflammation has only recently emerged (110, 111). Of the different mucosal sites where RA could be initiated, the lung has been the most studied to date in RA. Lung disease, both airway and parenchymal, and lung-generated autoimmunity, can be present before the initiation of joint symptoms, suggesting that immune reactions in the lung may be involved in the onset of RA-related autoimmunity (110, 112–114). Airborne pollutants (such as textile dust, silica and cigarette smoke) increase epithelial permeability with the accumulation of inflammatory mediators (115, 116).

While the particular immunologic pathways occurring in the lung prior to the onset of RA have yet to be entirely elucidated, data support the hypothesis that autoantibody development is initiated in the lung. Willis et al. demonstrated the presence of ACPA/RF in the sputum of patients with early RA, as well as in healthy controls with a family history of RA and/or ACPA positivity, with higher ratios of autoantibody in sputum than in serum (117). In this study, at least one RA-related antibody was identified in the sputum in 86% of early RA subjects and in 65% of seropositive at-risk subjects. Importantly, 39% of seronegative at-risk individuals also had sputum positivity for at least one RF/ACPA, supporting the contention that RA-related antibodies can be locally generated in the lung. These findings suggest that the lungs not only participate in the citrullination of proteins, but also that they are either an early target for injury secondary to autoimmunity or that they play a role in its development (117).

Demoruelle et al. studied early RA patients and at-risk subjects with autoantibody positivity for signs of airway abnormalities using high-resolution CT (114). Signs of inflammation, such as bronchiectasis, bronchial wall thickening and centrilobular opacities, were common in at-risk individuals and were similar to the airway abnormalities seen in patients with early RA. Similarly, Joshua et al., examined 106 patients with recently diagnosed untreated RA and found that the presence of RF and ACPAs, and high number of ACPAs specificities were associated with pulmonary parenchymal abnormalities (118). These findings support an important pathogenic link between systemic autoimmunity and the lung, contributing to RA development. Scher et al. demonstrated that local pulmonary and systemic autoimmune generation in untreated, recent-onset RA is associated with characteristic bronchoalveolar taxa, similar to sarcoidosis patients, but different from healthy controls (119).

Additional data also suggest that mucosal (lung mucosa included) processes play a role in the transition from pre-RA to classified RA. In a cohort of 214 subjects with serum samples obtained pre- and post-RA diagnosis, Kelmenson et al. demonstrated that the IgA isotype of ACPA significantly increased in positivity during a time period immediately prior to diagnosis of RA and shortly after, while IgG-ACPA positivity remained stable (120). Although this finding does not directly involve the lung, it suggests that IgA-dominant mucosal processes likely help the transition to clinically apparent inflammatory arthritis.

Furthermore, there is evidence suggesting that smoking may be related to the translocation of supraglottic bacterial species, such as Porphyromonas and Prevotella, to the lungs, thereby leading to increased peptidylarginine-deiminase (PAD) activity and airway inflammation (121). Taken together, these findings support the hypothesis that changes in the pulmonary microbial/host interface might contribute to RA pathogenesis. This is in line with previous studies involving early RA subjects that reported dysbiotic states at other mucosal sites, such as the gut and gingiva, with decreased taxa diversity (122).

If autoimmunity in RA originates in the lung, additional pathways must be involved in the transition from mucosal inflammation to the ultimate joint inflammation. Mechanisms that have been proposed include a epitope spreading to a joint specific antigen, shared antigenic target between the lungs and joints and immune complex deposition in the joints that drives finally to RA development (123).

To conclude, there is some evidence that the lung may be an early site of autoimmune-related injury and/or a potential site generating RA-related autoimmunity. More studies are needed to entirely understand the relationship between mucosal processes, as well as joint disease in RA, from the early triggering and dissemination of autoimmunity to the ultimate development of joint disease.

### Periodontal Disease

It is worth noting the great interest in recent years surrounding *Porphyromonas gingivalis* as a possible stimulus for the development of RA. This is because periodontitis (PD), is a disease that is more frequent in subjects with RA than in healthy populations (124), and *P. gingivalis* is the main causative agent of PD. It is the only bacterium known to express the PAD enzyme, which is responsible for the process of protein citrullination (125). This can lead to the generation of self-antigens, thereby producing chronic inflammation, characterized by the presence of pro-inflammatory cytokines and TNF (126). Several studies have shown that the prevalence of PD is higher in RA patients, in pre-RA patients and in FDRs of RA patients (49, 127, 128). Indeed, Cheng et al. demonstrated that anti-CCP positive at-risk subjects have dysbiotic subgingival microbiomes and increased abundance of *P. gingivalis* compared with healthy controls (129). Mikuls et al. described that anti-*P. gingivalis* concentrations have been significantly associated with autoantibody-positive and high-risk status of developing RA (130). There is a characteristic alteration in the composition of salivary microbes in individuals at high-risk for RA, suggesting that oral microbiota dysbiosis occurs in the “pre-clinical” stage of RA (131–133). Moreover, among patients with RA, PD severity appears to independently predict RA severity (134, 135). Together, these findings suggest that infection with *P. gingivalis* might lead to the expression of citrullinated neo-antigens, which, in genetically predisposed individuals, could lead to mucosal-based immune responses that finally trigger RA.

### Smoking

Smoking is the most important environmental risk factor for RA development. Several theories have been put forward as to how smoking might predispose individuals to RA. Smoking leads to higher expression of the PAD2 enzyme, that increase the mechanisms of citrullination in the lung (136). However, it remains unclear how tolerance against citrullinated proteins is broken and ACPAs are produced, since citrullination also occurs under physiological conditions. The association between smoking and RA presents an OR > 2, and smoking is estimated to account for approximately 20–30% of the environmental risk for RA (137). The relationship between smoking and RA present several aspects that contribute to an increased risk of developing RA. First, smoking is associated to a greater extent with ACPA-positive RA, especially in relation to shared epitope (SE) (137). Smoking has also been associated with the presence of RF even in the absence of RA (138). This suggests that there may be biological interactions between RA-related autoimmunity and the development of RA. Second, because smoking is associated with increased PD and lung, this risk factor may drive inflammation and alterations in local immunity. On the other hand in an FDR study of RA patients, joint inflammation, and tenderness was associated with smoking even in the absence of RA-related autoantibodies. This raises the possibility that smoking, in addition to its systemic effects, may also have direct joint effects that may be related to the future onset of IA (38). Furthermore, several epidemiological studies have shown that smoking appears to be a greater risk factor for RA in men than in women (139, 140). This observation could indicate that there are sex-related differences in the effects of smoking, or that women have other stronger risk factors for developing RA.

However, the question is where to place smoking in the context of RA onset. Really, it is unclear whether exposure to smoking triggers the initial autoimmunity or if it drives the propagation of autoimmunity. Some studies suggest that the effects of smoking possibly arise after the initial generation of RA-related autoimmunity, and may be related to prolonged “high-dose/intensity” smoking, as measured in pack-years. Other studies, however, suggest that it is the duration of smoking, more than the intensity of it, that amplifies risk of RA (51, 141, 142).

Furthermore, tobacco can interact with other risk factors.A prospective study involving 55 ACPA-positive individuals at risk for developing RA showed that smoking and being overweight increased the risk of development of arthritis (107) after 13 months of follow-up. So, these results show the importance of lifestyle factors in the development of RA.

To conclude, smoking is an important risk factor associated with the onset of RA, mainly in ACPA-positive individuals. However, this topic needs to be considered in future clinical research aimed at disease prevention as a modifiable risk factor.

### Silica/Dust Inhalation

Multiple studies have demonstrated an association between exposure to occupational silica/dust and RA, mainly in ACPA-positive RA (143–145). Indeed, “a study of firefighters and other emergency responders exposed to dust at the site of the 2001 World Trade Center collapse in New York, United States, found an increased risk of systemic autoimmune diseases, including RA” (145). The dust contained silica, pulverized cement, glass fibers, asbestos, and other materials. In other cohort of Malaysian women, occupational exposure to textile dust was also found to be significantly associated with an increased risk of developing RA (114).

In addition, the exposure to inhaled particles from air pollution, have also been linked to an increased risk for RA. However, it is difficult to assess the true exposure to air pollution and accounting for other factors such as specific components of pollution that may vary by locality (146, 147).

### Viral Infections

Several infectious agents have been implicated in the development of RA based on a higher frequency of positive viral serologies or their presence in the fluid synovium of RA patients. In addition, previous studies show that RA exhibits seasonal tendencies, suggesting the influence of viral infections on the onset of RA. However, their role as an agent for disease remains controversial (148). Pathogen-based infections such as the Epstein-Barr Virus (EBV), Proteus, Mycoplasma, coronavirus, ambient parainfluenza, and metapneumovirus have been associated with an increased incidence of RA (149). Few studies have investigated the potential link between respiratory viral infections and the development of RA (150, 151). In a population-based case-control study, previous respiratory tract infections treated with antibiotics (sinusitis, tonsillitis and pneumonia), showed no association with an increased risk of RA (150). Another study, showed that viral infection symptoms confirmed by questionnaire were more frequent in patients who had experienced a new-onset RA during the previous year compared to healthy controls, although this was a small-sized study involving 59 RA patients and 69 controls (151). In a national cohort study, it was observed that individuals hospitalized for a serious infection were at increased risk for a subsequent diagnosis of autoimmune diseases, including rheumatoid arthritis (152). Individuals with multiple hospitalizations for infections and persons hospitalized for a serious infection shortly before an autoimmune diagnosis were at greatest risk, and no differences were found between being hospitalized for a viral infection and other types of infection. This study suggests that all infections requiring hospitalization increase the risk of developing an autoimmune disease regardless of the type of infection, albeit in a time-dependent manner. However, rather than directly initiating autoimmunity, serious infections might accelerate a pre-existing autoimmune condition to progress to clinical disease.

Viral arthritis is distinct from autoimmune disease-associated polyarthritis, and a differential diagnosis should be performed. Viral arthritis are usually self-limiting, and treatment with immunosuppressants is not required in most cases. However, arthritis associated with HIV, HCV, Lyme disease, and gonococcal arthritis may follow a chronic development, mimicking RA.

In summary, infectious agents may play some role in the development of the disease in the context of genetic predisposition and not in isolation, but rather by interacting with other risk factors.

## Serological and Immunological Factors

### Auto-Antibodies

The presence of different auto-Abs in serum can be detected years before RA disease onset (3, 153, 154). Several longitudinal studies have linked the presence of ACPA to the onset of clinical arthritis (8, 155–157). In fact, the value of ACPA levels in predicting arthritis development is unclear. Two studies supported an association between ACPA levels and arthritis development (21, 155) while two others did not (93, 157). In addition, the number of epitopes recognized by ACPA was associated with the onset of arthritis in several studies involving ACPA-positive patients with arthralgia (158–160). Furthermore, a decrease in galactosylation and an increase in the core fucosylation of serum ACPA IgG1 prior to the RA onset has been reported, indicating that these autoantibodies adopted a more inflammatory phenotype (161). In addition, clinical differences between ACPA positive and ACPA negative patients with CSA in the symptomatic phase preceding clinical arthritis has been described (162). On the other hand, it is worth noting that the effect of ACPA on the risk of developing RA can be potentiated by its association with other risk factors such as obesity (106).

The value of RF in the preclinical phase of RA has also been studied (8, 21, 155–157). Two studies, using the same cohort, demonstrated the additive presence of RF in association with arthritis development in ACPA-positive patients (13, 155). No ACPA-negative patients were included in the previously mentioned studies. Other studies lost the single value of the RF-positive results after adjusting for the concomitant presence of ACPA (8, 156, 157). However, the results from one study suggested that high (vs. low) levels of RF are a predictor of RF (157).

Anti-carbamylated protein (anti-CarP) antibodies also belong to the group of anti-post-translationally modified protein antibodies (AMPA) that have been described in RA. The relationship between the presence anti-CarP antibodies and the risk for RA development during the preclinical phase of RA remains controversial. Two studies found an association between anti-CarP antibodies and the development of RA in patients with arthralgia (163), whereas another study in patients with CSA found no additive value of anti-CarP when ACPA and RF status is known (157). One study conducted in 124 FDRs observed that the presence of anti-CarP antibodies (anti-FCS-Carp, anti-Ca-Fib3, and anti-Ca-Fib2) are more frequent in FDRs than in health controls (164). In addition, the presence of these antibodies were associated with other non-SE alleles suggesting that additional mechanisms may be influencing the risk of developing RA (164).

The role of other RA related auto-Abs (anti-acetylated protein Abs, anti-BRAFF, anti-PAD4 or Anti-oxidized protein antibodies) prior to RA onset has not been evaluated.

In conclusion, the presence of ACPA is associated with arthritis development, although this is less clear for RF and anti-CarP antibodies. Nonetheless, it is important to highlight the fact that the available evidence on this issue poses some limitations: the absence of an auto-Ab negative reference group, the lack of a standard definition of arthralgia in the inclusion criteria, and some studies involved the same cohort.

### Acute Phase Reactants

Several acute phase reactants, chemokines, cytokines, and other systemic markers have been studied in the preclinical stages of RA (11). Results of studies evaluating CRP and the erythrocyte sedimentation rate (ESR) are controversial. Some studies identified an association between CRP or ESR and arthritis development (119, 161), while others did not (19, 155, 160, 165–167). The only study that showed an association between serum CRP at study entry and development of arthritis included patients with CSA and did not select on the presence of autoantibodies (21, 162). Studies showing no predictive value for CRP levels were mostly conducted in patients with autoantibody-positive arthralgia (19, 155, 160, 165–167). This implies that CRP has a predictive value, particularly, in autoantibody-negative patients.

In conclusion, most results on serological markers of inflammation have not been validated in independent studies. Only CRP has been evaluated in different patient cohorts presenting seropositive arthralgia, although it was shown to be of limited value.

## Future Perspectives

There are many clinical, analytical, sociodemographic and environmental risk factors that have been associated with the risk of developing RA. The early identification and prevention of chronic diseases such as RA has aroused great interest in the scientific community due to their far-reaching implications. In this review, it is observed that the risk factors associated with the onset of RA are very heterogeneous, some of them are modifiable. This highlights the importance of educating citizens about the importance of healthy lifestyle habits in order to prevent chronic diseases.

One of the main hurdles is that the risk factors associated with RA are exacerbated by the interaction between them, which makes it quite difficult to determine the individual risk levels of each individual within a given period of time. Concerning this issue, and the amount of variables to take into account, new techniques and research methodologies such as “Big Data” analytics and artificial intelligence, may set the groundwork for deeper predictive insights. To this end, they may allow us to formulate more accurate predictive models than those described thus far.

On the other hand, it is to clarify if the risk factors are for auto-antibodies development and/or for the clinical onset of the disease and how their interaction with the genetics or epigenetics of the patient predispose to develop RA. Understanding these mechanisms in depth will help us to improve our knowledge about the development of the disease and to define additional risk factors that could be included in predictive models.

Other crucial aspects to clarify are whether the identification of these at-risk individuals requires specific diagnostic and/or therapeutic strategies, and the real impact of these preventive actions in both the short- and long-term.

## Conclusions

Several studies have attempted to identify risk factors in patients presenting early UA and CSA, with a particular focus on clinical and serological criteria inherent to the individual. However, there are many potentially modifiable factors that can be taken into account during this early stage of the disease, not only in terms of identifying patients at an increased risk of developing RA, but also as to whether patient care should be initiated in order to prevent disease progression. It should be kept in mind that these factors do not act alone, and that the combination of several factors results in a heightened risk of RA. Thus, it would be very useful to implement the knowledge gained about these factors into a more comprehensive management plan for the care of such patients.

## Author Contributions

All authors designed, developed, and contributed to the final manuscript. All authors read and approved the final manuscript.

## Conflict of Interest

AB received grant/research support from Abbvie, Pfizer, Novartis, BMS, Nordic, Sanofi, Sandoz, UCB, Roche. Worked as a paid consultant for Abbvie, Pfizer, Novartis, BMS, Nordic, Sanofi, Sandoz, Lilly. Has been paid as a speaker for Pfizer, Novartis, UCB, Nordic, Sanofi, Sandoz, Lilly. CP-R has received research grants/honoraria from AbbVie, Lilly, Novartis, Pfizer, Sanofi, Biogen, and UCB. The remaining authors declare that the research was conducted in the absence of any commercial or financial relationships that could be construed as a potential conflict of interest.
